# Double‐digest RADseq loci using standard Illumina indexes improve deep and shallow phylogenetic resolution of *Lophodermium*, a widespread fungal endophyte of pine needles

**DOI:** 10.1002/ece3.4147

**Published:** 2018-06-11

**Authors:** Rodolfo Salas‐Lizana, Ryoko Oono

**Affiliations:** ^1^ Department of Ecology, Evolution, and Marine Biology University of California Santa Barbara California; ^2^ Present address: Departamento de Biología Comparada Facultad de Ciencias Universidad Nacional Autónoma de México Mexico City Mexico

**Keywords:** ddRADseq, genotyping by sequencing, sequencing depth

## Abstract

The phylogenetic and population genetic structure of symbiotic microorganisms may correlate with important ecological traits that can be difficult to directly measure, such as host preferences or dispersal rates. This study develops and tests a low‐cost double‐digest restriction site‐associated DNA sequencing (ddRADseq) protocol to reveal among‐ and within‐species genetic structure for *Lophodermium*, a genus of fungal endophytes whose evolutionary analyses have been limited by the scarcity of informative markers. The protocol avoids expensive barcoded adapters and incorporates universal indexes for multiplexing. We tested for reproducibility and functionality by comparing shared loci from sample replicates and assessed the effects of numbers of ambiguous sites and clustering thresholds on coverage depths, number of shared loci among samples, and phylogenetic reconstruction. Errors between technical replicates were minimal. Relaxing the quality‐filtering criteria increased the mean coverage depth per locus and the number of loci recovered within a sample, but had little effect on the number of shared loci across samples. Increasing clustering threshold decreased the mean coverage depth per cluster and increased the number of loci recovered within a sample but also decreased the number of shared loci across samples, especially among distantly related species. The combination of low similarity clustering (70%) and relaxed quality‐filtering (allowing up to 30 ambiguous sites per read) performed the best in phylogenetic analyses at both recent and deep genetic divergences. Hence, this method generated sufficient number of shared homologous loci to investigate the evolutionary relationships among divergent fungal lineages with small haploid genomes. The greater genetic resolution also revealed new structure within species that correlated with ecological traits, providing valuable insights into their cryptic life histories.

## 1 INTRODUCTION

The genetic diversity and structure within species in combination with geographical and ecological metadata can uncover important biogeographical structuring, demographic history, and adaptive traits linked to ecological functions (Loveless, 1984; Manel, Schwartz, Luikart, & Taberlet, 2003; Neale & Savolainen, 2004; Slatkin, 1987). The genetic relationship among closely related species, or phylogenetic associations, can also uncover important life history, such as ecological events or traits that influence diversification and speciation (Huyse, Poulin, & Théron, 2005). Microbial species, whose life histories and species delimitations are particularly challenging to observe or measure, would especially benefit from genetic sequencing methods that can uncover both micro‐ and macroevolutionary diversity. In this study, we test a low‐cost double‐digest restriction site‐associated DNA sequencing (ddRADseq) protocol that is commonly applied for population‐level SNP discoveries but also increasingly applied for phylogenetic analyses, on a fungal genus whose life cycle is understudied. *Lophodermium* (Chevall.) is a paraphyletic genus with over 100 named species that associate with dead or living plants worldwide (Lantz, Johnston, Park, & Minter, 2011). Those species associated with pine needles consist of a smaller (ca. 30 putative species) and closely related group (Ortiz‐García et al., 2003) that often dominate over other endophytic species (Ganley, Brunsfeld, & Newcombe, 2004; Oono, Lefèvre, Simha, & Lutzoni, 2015). Their ecological significance with pines is unknown beyond their “endophyte” status, although a few are considered pathogenic (Hanso & Drenkhan, 2011) and some are explicitly studied as saprophytes (Osono & Hirose, 2011). *Lophodermium* species also appear to have large within‐species genetic variation (Deckert, Hsiang, & Larry Peterson, 2002; Salas‐Lizana, Santini, Adán, & Piñero, 2012) with the occurrence of many cryptic species (Oono et al., 2014; Reignoux, Green, & Ennos, 2014). While a handful of genetic markers may successfully delimit populations or species to reveal dispersal rates or demographic histories, the population structure of some widespread species has yet to be identified with traditional multilocus sequence‐typing approaches (Oono et al., 2014; Salas‐Lizana et al., 2012).

High‐throughput sequencing allows the rapid generation of genomic data for hundreds of individuals to address diverse ecological and evolutionary questions. As a genotyping technique that can sequence more individuals for fewer loci, RADseq has become the most widely used, cost‐effective method, particularly for nonmodel species without reference genomes (reviewed in Andrews, Good, Miller, Luikart, & Hohenlohe, 2016; Davey et al., 2011). The double‐digest RADseq (ddRADseq; Peterson, Weber, Kay, Fisher, & Hoekstra, 2012), a variation of the original method (Baird et al., 2008), improves on depth of coverage per locus by optimizing sequencing effort and reducing missing genotypes. The protocol is flexible, allowing for easy optimization for different organisms, genome sizes, genetic diversity, and scientific questions (Mastretta‐Yanes et al., 2015; Nieto‐Montes de Oca et al., 2017; Recknagel, Elmer, & Meyer, 2013; Zhou et al., 2014). Consequently, numerous modifications or improvements of ddRADseq are being continually proposed (Franchini, Parera, Kautt, & Meyer, 2017; Heffelfinger et al., 2014; Recknagel, Jacobs, Herzyk, & Elmer, 2015). In this study, we evaluate an underutilized version of the popular ddRADseq protocol that accommodates the less‐expensive and standard indexed primers for multiplexing (Kess, Gross, Harper, & Boulding, 2016).

**Figure 1:**
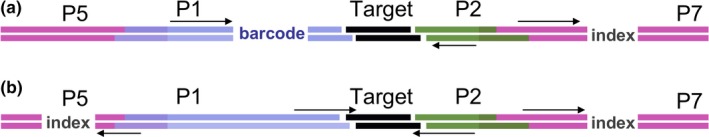
Design comparison between barcoded adapters (a) and the adapters in this study compatible with standard dual indexes (b). Arrows indicate sequencing primer regions. Sequencing primers for the target region are custom designed in (b) to overlap with enzyme cutting sites. P1 and P2 are adapters ligated to target fragments that may (a) or may not (b) have barcodes. P5 and P7 are flow cell‐binding regions for Illumina platforms and are always incorporated using PCR to the ligation adapters. See Appendix S2 for sequences of adapters and primers and cost comparison analyses

**Figure 2:**
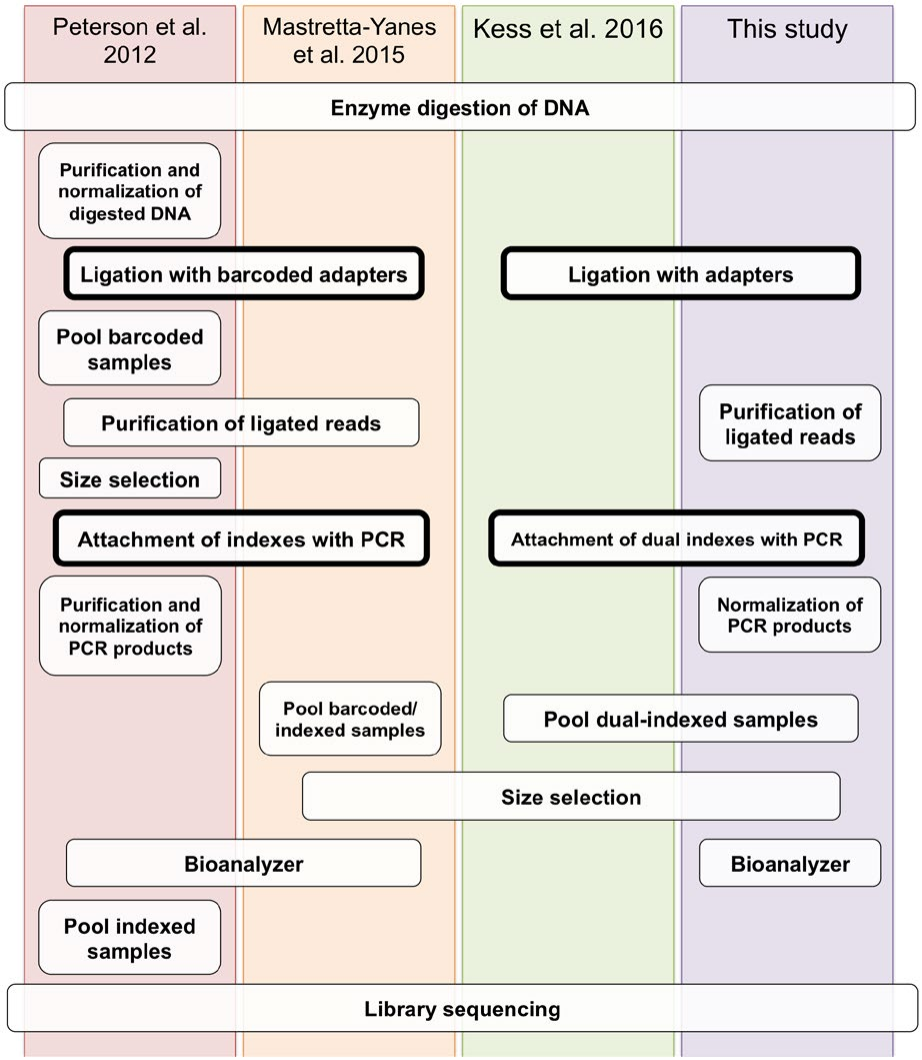
A comparison of four ddRADseq protocols. Optional steps are excluded. Detailed protocol for this study can be found in Appendix S2

Double‐digest RADseq consists of digestion of the genomic DNA with two different restriction enzymes (typically one with high‐frequency site and one with low‐frequency site), ligation of digested fragments to adapters, a PCR step for the enrichment of ligated fragments and attachment of indexes, size selection, pooling of samples, and finally, sequencing of pooled fragments using a high‐throughput sequencing platform. Multiplexing for ddRADseq is typically achieved by ligating adapters with unique in‐line barcodes to digested genomic DNA and then adding unique indexes during PCR enrichment (Figure 1 & 2; Parchman et al., 2012; Peterson et al., 2012; Mastretta‐Yanes et al., 2015). Barcoded ligation adapters need to be synthesized in pairs and can be pricey (e.g., $140 per pair), are specific to a restriction enzyme, and can therefore only be applied with ddRADseq. For example, preparing a 96‐sample ddRAD library with eight barcoded adapters and twelve indexes requires an investment of over $1,000 in adapters alone ([eight forward + one reverse] × 2 complementary annealing oligos × $70 = $1,260) that cannot be used for non‐ddRAD sequencing projects (unlike standard indexes). Consequently, the cost of barcoded adapters may impede the start of new projects for smaller laboratories. In this study, we modified protocol that was previously used for SNP discovery in a single species by Kess et al. (2016) that lowers the upfront costs from barcoded adapters and allows the use of commonly applied combinatorial dual‐indexed barcodes using standard adapters compatible with cohesive restriction enzyme sites, and sequencing using low‐cost custom primers that include the restriction site. See Appendix S2 for detailed cost comparisons to original ddRAD protocol. We explored the reproducibility and genetic diversity revealed by the low‐cost ddRADseq protocol on multiple species of the genus *Lophodermium*.

At present, Illumina HiSeq is the most commonly used platform for genome resequencing albeit the short read lengths per loci (e.g., <100 bps; but MiSeq is used by Davik et al. (2015), Kess et al. (2016) and Vivian and Rn (2017)). This platform has the ability to produce sufficient coverage depth per locus, given an appropriate multiplexing density, to overcome potential sequencing errors and false SNP calls as well as produce high numbers of shared loci among samples. The deep coverage can be particularly crucial for identifying heterozygosity in nonhaploid organisms. We tested the protocol on the MiSeq platform instead because we believed fewer sequence reads would still produce sufficient numbers of shared loci among samples for population genomic and phylogenetic analyses, given the relatively small haploid genomes of these species (est. 40–80 Mbps). We were also interested in understanding how sequencing depth affected the ability to identify shared loci or recover loci repeatedly across samples with the longer reads (e.g., >100 bps). We assessed the recoverable number of shared loci among technical replicate samples by determining the relationship between the number of shared loci between replicates and their sequencing depths. Furthermore, because the coverage per locus would be comparably lower than in HiSeq, potentially introducing misidentified variants into the final dataset from errors during sequencing, PCR, or other technical modifications in the preparation of the library, we also compared the error rates between shared loci in technical replicate samples run either within or between Illumina lanes.

The purpose of this study was twofold: (i) constructing a robust phylogeny of a genetically diverse group of understudied fungi using a rapid and low‐cost ddRADseq protocol with standard indexes and (ii) assessing the relationship among clustering and filtering parameters, error rates, and numbers of homologous loci for micro‐ and macroevolutionary analyses. We also evaluated the recoverable number of shared loci with increasing genetic divergence within *Lophodermium* and how these loci might improve species circumscription compared to the widely used internal transcribed spacer (ITS) rDNA locus. We demonstrate its utility on 50 fungal isolates representing populations of six putative species based on ITS phylogenetics. We show that this low‐cost ddRADseq protocol applied on the MiSeq can be effectively implemented for circumscribing more putative fungal species and finer population structures than ITS alone. We demonstrate its potential to significantly improve phylogenetic resolution for nonmodel organisms and reveal population structure within widespread species, but clustering and filtering are still key bioinformatic stages that need to be tested for different groups of organisms.

## 2 MATERIALS AND METHODS

### 2.1 Sampling, culturing, and DNA extraction

Multiple *Lophodermium* species and individuals were collected from both ascocarps on senescent needles and mycelial cultures from healthy green needles of *Pinus* spp. (see Table S1A for geographic origins, host species, and isolation methods). Monosporic cultures were obtained from ascocarps as described in Salas‐Lizana et al. (2012) using 2% malt extract (ME) agar. The *Lophodermium* isolates from green needles used in this study come from an ongoing survey of endophytes of pine trees. Green needles were washed and surface‐sterilized as described in Oono et al. (2015). Green needles were cut with a sterile razor blade into 2‐mm‐long sections. Sections were placed in 2% ME agar slants in 1.5 ml microcentrifuge tubes in sterile conditions. A subset of emerging cultures was genotyped by sequencing the internal transcribed spacer (ITS) and partial large subunit (LSU) rDNA region (hereafter ITS‐LSU) for positive identification of a *Lophodermium* spp. Several samples come from the fungal collection at New Zealand Crown Research Institute (Scion). With the exception of three species (*L. australe*,* L. conigenum*, and *L. pinastri*), the remaining specimens were identified using a combination of ascocarp morphology (data not shown) and ITS‐LSU sequences. The ITS‐LSU sequences of *L. australe* and *L. conigenum* were similar enough (>97% sequence similarity) to be considered a single putative species in this study, which is also suggested by others (Minter, 1981; Ortiz‐García et al., 2003). Cultures were grown for 2–3 weeks in 50 ml of 2% ME media and dried on filter paper under sterile conditions. Between 40 and 100 mg of dried mycelium was used for DNA extraction following a modified CTAB method, see Appendix S1 for details. DNA quality was assessed by visualizing on 1.5% agarose gels, confirming the absence of RNA/DNA smears. DNA quantification was performed on a Qubit 2.0 fluorometer using the Qubit dsDNA HS Assay Kit (Invitrogen, Carlsbad, CA, US).

### 2.2 ITS‐LSU sequencing and phylogenetic analyses

We amplified the ITS‐LSU rDNA region (ITS1, 5.8S, ITS2, and partial 28S) using primers ITS1F (Gardes & Bruns, 1993) and LR3 (Hopple & Vilgalys, 1994). PCR protocol consisted of an initial denaturation step at 95°C for 4 min, followed by 35 cycles of 30 s at 95°C, 30 s at 50°C, and 90 s at 72°C, and a final extension at 72°C for 10 min. PCR products were cleaned with ExoSAP‐IT (USB‐Affymetrix, Santa Clara, CA) and sequenced using Sanger technology at UC Berkeley DNA Sequencing Facility. The ITS‐LSU sequences were aligned using MAFFT v7 (Katoh & Standley, 2013) with default parameters for the 50 sampled *Lophodermium* individuals alone as well as with 55 additional reference ITS‐LSU sequences from public databases (see Table S1B for ITS accessions). We compared the topologies of unrooted trees constructed by ITS‐LSU alone and all ddRAD loci. In all cases, trees were obtained using RAxML v8.0.26 (Stamatakis, 2014) with 1000 bootstrap replicates under the GTRGAMMA model (Tavaré, 1986).

### 2.3 ddRADseq library preparations

Three to nine hundred nanograms of DNA per sample (6 μl total; 50–150 ng/μl) was double‐digested using the rare‐cutting EcoRI‐HF and the frequent‐cutting MseI enzymes (New England BioLabs, Ipswich, MA). In cases where starting DNA concentrations were low, duplicate samples underwent digestion and ligation steps and then were pooled (see detailed workbench protocol for additional tips and similarities with Kess et al. (2016) in Appendices S1–S2). All samples were placed randomly in PCR plates during library preparation. The DNA was digested for four hours at 37°C, and the enzymes were heat‐killed at 65°C for 10 min. Digested DNA fragments were ligated to the EcoRI‐specific P1 adapter and the MseI‐specific P2 adapter (Figure 1b, Appendix S2), which are not barcoded as in the original ddRADseq protocol (Peterson et al., 2012), with the T4 ligase enzyme (New England BioLabs, Ipswich, MA). The ligation reaction consisted of incubating overnight (>12 hs) at room temperature (approx. 21°C) and heat killing the enzyme at 65°C for 10 min. In order to eliminate unincorporated adapters and small (<300 bps) DNA fragments, ligation reactions were purified using Agencourt AMPure XP SPRI magnetic beads (Beckman Coulter, Brea, CA) at 0.8:1 (beads:sample) volume ratio and resuspended in 35 μl of 10 mmol/L Tris buffer. A unique combination of the Illumina Nextera v2.0^®^ dual‐indexed barcodes (P5 and P7; Illumina, Inc. San Diego, CA) was attached to purified fragments with 14 cycles of PCR for two replicates of each sample. Indexed PCR products were normalized and pooled in equimolar proportions. Fragment sizes between 300 and 700 bps were selected using Pippin Prep (Sage Science Inc., Beverly, MA) 2% agarose cassettes. The final libraries were analyzed using a TapeStation Instrument (Agilent Technologies, Santa Clara, CA) to confirm the recovery of fragments on the selected range (see Appendix S2 for fragment size profiles). The library concentrations were quantified as previously described and sequenced using Illumina MiSeq platform under a 251‐cycle paired‐end read protocol at the IGM Genomics Center, UC San Diego. In total, four libraries, which included samples from other studies, were sequenced. See Table S1C for library description and barcoded samples.

We tested reproducibility of the ddRAD data by sequencing technical replicates of six *Lophodermium* strains within and among libraries. Replicates are samples from the same genomic DNA that were processed independently (i.e., separate enzyme digestion) and tagged with different combinations of P5 and P7 indexes (see Table S1C for details).

With traditional Illumina sequencing primers, the sequencer detects zero sequence diversity at the beginning of these libraries because they all have identical restriction site patterns. This initial low‐diversity impairs the MiSeq system to “maintain focus, register images to the cluster map, and make proper base calls to deliver high‐quality data (Pub. No. 770‐2013‐013, Illumina technical support note).” Hence, custom sequencing primers were used that include the restriction site bases (Figure 1b; Appendix S2), which allows the sequencer to detect high sequence diversity from the beginning. These primers also allow the user to maximize sequencing the variable loci and to avoid sequencing the barcodes or restriction sites (Figure 1; Kess et al., 2016).

### 2.4 Bioinformatics—filtering and clustering ddRADseq

Paired‐end reads were demultiplexed by the sequencing facility and merged using PEAR (Zhang, Kobert, Flouri, & Stamatakis, 2014) with default parameters: a minimum overlap of 10 bps, a minimum assembled sequence length of 50 bps, and a maximum p‐value of .01 for the observed‐expected alignment score, which tests statistical significance of merged reads. We excluded unassembled pairs since, on average, <1.58% of reads were unmerged (Table S1F, G), suggesting that analyzing merged reads with lower sequencing errors was more valuable to us than retaining longer reads with lower likelihood of identifiable shared homology across samples.

Merged reads were processed using pyRAD v3.0.66 pipeline (Eaton, 2014) to filter for quality and potential paralogs (more detail below). Nucleotide bases with Phred Q‐scores <20 (i.e., accuracy of the base call is <99%) were changed to an “N” character and reads were excluded (i.e., quality‐filtered) based on the allowable number of Ns per read. We tested a range of allowable number of Ns per read from 5 to 30 (Table S1E), which represents approximately 1.8%–10.7% of nucleotides per read given an average assembled sequence length of 281 bps (Table S1F). The default of 4 Ns (appropriate for short HiSeq reads) would have been too stringent for the longer MiSeq reads. The quality‐filtered reads were then clustered within samples using 70% or 85% sequencing similarity thresholds. We analyzed the effect of the number of allowed Ns during the filtering step on the number of clusters per sample.

Filtered reads were clustered within samples using VSEARCH v1.11.1 (Rognes, Flouri, Nichols, Quince, & Mahé, 2016). We tested a range of clustering thresholds between 70 and 95% sequence similarity at 5% increments for samples that were filtered for reads with a maximum of 15 Ns (hereafter described as “15N”). We then analyzed the effect of clustering thresholds on the average coverage depth (i.e., number of reads per cluster) per sample. We also analyzed reads filtered with a maximum criteria of 30 Ns (i.e., “30N”), rather than 15N, and clustered these reads just at 70% sequence similarity (hereafter described as “70/30N”) to include in our comparative analysis. Putative loci or clusters represented by only one read (i.e., singletons) were discarded.

A consensus sequence is called for each putative locus or cluster using either the estimated error rate or the majority rule. When a cluster is represented by more than three reads, consensus base calls are made using only A/T/G/C or N based on a sequencing error rate that is estimated by maximum likelihood across all clusters (i.e., putative loci) within a sample, assuming zero heterozygosity because fungal DNA was haploid. Any ambiguous base site that occurs more often than the expected rate based on the estimated error rate is called “N.” On the other hand, when a cluster is represented by only two reads, consensus base calls are made using the majority rule, which chooses the lower alphabetical base to call ambiguous bases (i.e., A/T = A; A/G = A; A/C = A; and G/C = C). The majority rule base call introduces bias in the data (e.g., more likely to share As at the same site than Ts), but allows retention of low coverage loci. Loci with consensus sequences with more than five Ns are discarded, which helps to remove potential paralogs. Decreasing clustering similarity (i.e., from 85% to 70%) increases coverage per cluster, but can also increase potential paralogs within clusters as well as SNP error rates.

These loci were then clustered among samples using VSEARCH again and aligned using MUSCLE v3.5 (Edgar, 2004). Aligned loci were kept in the final dataset if there were fewer than 100 SNPs and 99 insertions/deletions (indels) across samples (default values). These criteria discard longer reads (>330 bps) whose clustering threshold (e.g., 70%) produce highly variable loci. Because our data were already demultiplexed and paired reads were merged, the PyRAD pipeline was started in step two, where we set the datatype to *ddrad*, instead of *pairddrad*. Summary information for 85/15N and 70/30N pyRAD pipeline runs can be found in Tables S1F, G.

### 2.5 Phylogenetic analyses with ddRAD loci

To explore the usefulness of the longer ddRAD reads for inferences at a deep time scale (i.e., phylogenetics), we compared the genetic distance, measured using ITS‐LSU rDNA region, to the number of shared loci between pairs of 50 individuals from six putative species of *Lophodermium* (*L. australe‐L. conigenum* complex*, L. baculiferum, L. molitoris, L. nitens, L. pinastri,* and *L. sp. nov*.). Clustering thresholds between 55 and 70% sequence similarity are considered to reconstruct the most accurate phylogenetic topologies for recent species divergences (e.g., <60 million years) but is highly recommended to be tested at various clustering values (Rubin, Ree, & Moreau, 2012). As our reads are longer than typical ddRAD reads sequenced on the HiSeq, we tested clustering at 85% threshold (DaCosta & Sorenson, 2014; Heffelfinger et al., 2014) allowing 15 Ns as well as at a lower 70% threshold allowing 30 Ns. Hereafter, the final datasets using these two criteria are referred to as 85/15N and 70/30N matrices, respectively. Loci found in more than 10 samples were kept in the final dataset for phylogenetic reconstruction. We chose 10 as the minimum sample number per locus because the maximum sample size within a putative species was eight (with the exception of *L. nitens* and *L*. *sp. nov*., which are likely species complexes with multiple cryptic species). Therefore, a minimum sample of 10 would most likely result in the inclusion of more than one species for each locus in the final alignment. We compared the phylogenetic resolution between the 70/30N and 85/15N matrices. All phylogenetic analyses were performed using RaxML v8.0.26 (Stamatakis, 2014) as described above. We also evaluated the number of shared loci within species using the two criteria for species represented by at least eight individuals.

Pairwise genetic distances between samples and putative species were estimated using Kimura 2‐parameter model (Kimura, 1980) considering variable rates among sites (α = 0.5) in the ITS‐LSU locus after testing several evolution models using a maximum‐likelihood criterion (lnL) in MEGA v7 (Kumar, Stecher, & Tamura, 2016). The effect of genetic distances on the number of shared loci among pairs was analyzed by best‐fit models with minimum number of parameters in TableCurve 2D v3 (Systat Software Inc.).

### 2.6 Reproducibility analyses

We tested the reproducibility of our molecular bench protocol by estimating variation in base calls between nine technical replicate pairs (using six individual samples) within or between sequencing runs (Table S1D). We filtered the replicate pairs using both 70/30N and 85/15N criteria and calculated the proportion of variable sites between shared loci (i.e., number of SNPs divided by the total length of concatenated shared loci). The variable sites between shared loci of replicate pairs should represent the error rates introduced during PCR, sequencing, filtering, and clustering. We also tested how sequencing depths (i.e., total number of reads per sample) related to the total number of shared loci between replicate pairs after filtration. We were interested in understanding whether increasing sequencing depth has significant effects on the number of shared loci between replicate pairs or on the error rates.

## 3 RESULTS

### 3.1 ddRADseq data summary

A total of 11,901,179 sequence reads were analyzed for this study (including replicate samples) which were produced in four separate libraries consisting of 44, 48, 64, and 68 samples (Table S1C). Demultiplexed, raw sequence reads are available in the NCBI SRA under BioProject accession PRJNA35677. On average, 98.42% (*SD* 0.89) of all reads were merged with a minimum of 94.41% and a maximum of 99.41% per sample (Table S1F). The average number of merged reads per sample was 201,715 with an average length of 281 bps. The mean quality (Q) score of merged reads ranged from 35.70 to 39.60 for the first 251 nucleotide positions, 33.60 to 31 up to position 485, and then from 30.90 to 28.70 at final position 492. The drop in Q‐score is typical for reverse reads (Schirmer et al., 2015). The mean percentage of reads that passed quality‐filtering was 90.22% and 84.47% for 30N and 15N criteria, respectively. Within‐sample clustering recovered an average of 48,659 and 52,658 clusters per sample (*nloci*) for the 70/30N and 85/15N criteria, respectively. The mean depths per cluster, excluding singletons, were 8.16 (*SD* 11.30) for 70/30N and 7.71 (*SD* 11.20) for 85/15N, and correlated highly with sequencing depth per sample (Figure S1). The average percentages of *nloci* (i.e., represented by the consensus sequence of a given cluster) remaining after excluding singletons and those with more than five Ns in the consensus sequence (i.e., *f1loci*) were 28.26% and 30.03% for 70/30N and 85/15N criteria, respectively. The average percentages of loci remaining after excluding potential paralogs (*f2loci/nloci*) to compare across samples were 28.00% for 70/30N criteria and 26.71% for 85/15N. The average numbers of these remaining loci (i.e., *f2loci*) shared by a minimum of ten samples for phylogenetic analyses were 566 (3.87% of *f2loci*) for 70/30 criteria and 569 (4.04% of *f2loci*) for 85/15N. See Tables S1F, G for further details.

### 3.2 Allowable Ns at filtering stage and clustering similarity thresholds

**Figure 3:**
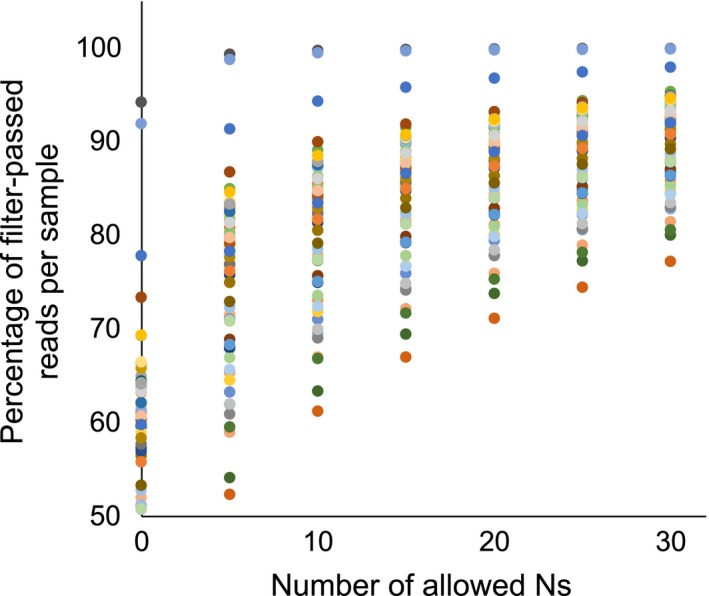
Percent filter‐passed reads vs. No. of allowed Ns per read. Each point represents a different sample filtered with different numbers of Ns. Nucleotide bases with Phred Q‐scores <20 were changed to an “N” character, and reads were excluded based on the allowable number of Ns per read. For example, excluding reads with more than five Ns is equivalent to excluding reads with more than 1.8% nucleotides per read on average with a Phred Q‐score less than 20, assuming an average read length of 281 bps. Excluding reads with more than 30 Ns is equivalent to excluding reads with more than 10.7% nucleotides per read on average with a Phred Q‐score less than 20, assuming an average read length of 281 bps

Increasing the allowed number of Ns per read from 0 to 30 Ns steadily increased the proportion of filter‐passed reads (Figure 3). On average, 43.2%, 15.5%, and 9.8% of reads had at least one, 15, and 30 Ns per sample, respectively. The marginal increase in filter‐passed reads decreased with each additional allowed number of Ns. See Table S1E for additional summary.

**Figure 4:**
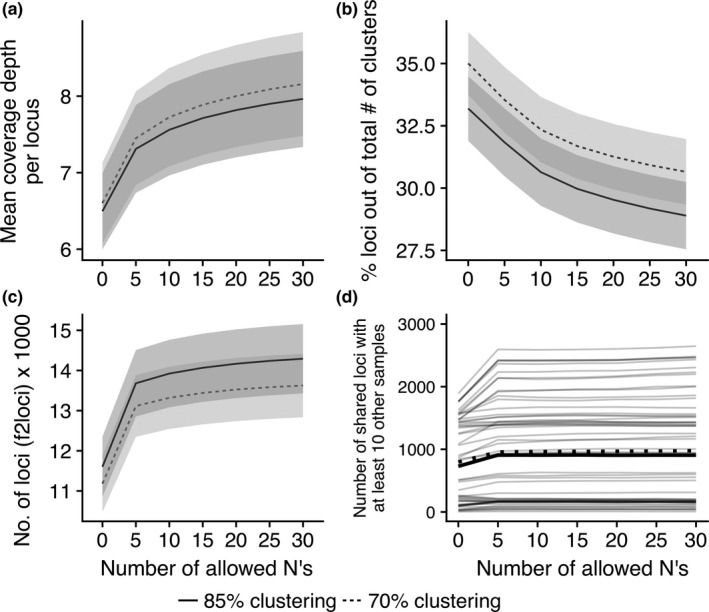
Effects of the number of allowed Ns in a filter‐passed read on the (a) coverage depth of clustered loci with more than one read, (b) percent loci (*f2loci*) out of total clustered loci (*nloci*) after filtering singletons and potential paralogs, (c) number of total loci (*f2loci*) per sample for two clustering threshold values (70% and 85%), and (d) number of loci shared (*f2loci*) by at least ten samples for 70% clustering threshold values for the 59 samples (light gray lines). Dotted and solid lines indicate average for loci clustered with 70% and 85% similarity, respectively. Gray areas represent standard error (*n* = 59). See also Table S1E for details

The number of clusters per sample increased on average with number of allowed Ns, regardless of clustering thresholds (Table S1E). When reads were clustered at 70% similarity, allowing zero, 15, and 30 Ns per read , these parameter combinations produced, on the average, 32,954, 45,999, and 48,659 clusters per sample, respectively. When reads were clustered at 85% similarity, allowing zero, 15, and 30 Ns per read, these parameter combinations produced, on the average, 37,124, 52,658, and 56 124 clusters per sample, respectively (see Table S1E, F, and G for summary). Increasing the number of allowed Ns also increased the average coverage depth per locus (Figure 4a), although the increase was minimal (i.e., <1) between 15 and 30 Ns. The final number of loci per sample to be compared across samples (i.e., *f2loci*) increased with increasing number of allowed Ns (Figure 4c). However, this increase was minimal from 15 to 30 Ns (e.g., 1.4% increase for 70% clustering and 1.6% increase for 85% clustering; Figure 4c). The quality‐filtering parameter had little effect on the average number of loci per sample used in the phylogenetic analyses when loci shared by fewer than 10 samples were excluded (Figure 4d).

**Figure 5:**
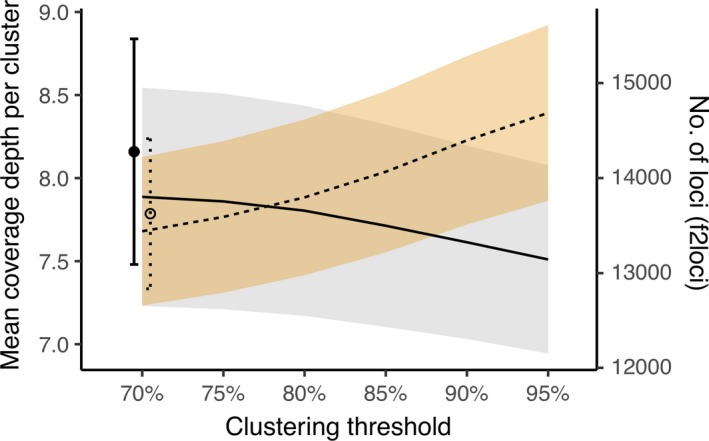
Effect of clustering thresholds on the mean depth per cluster and number of loci (f2loci), excluding singletons, per sample. Solid line indicates mean coverage depth (left axis) and dotted line indicates number of loci (right axis). In all cases, a maximum of 15 Ns was allowed in the quality‐filtering step. Shaded areas represent standard error (*n* = 59). Mean depth and number of loci for 70/30N are provided as a single point with standard error bars for comparison. See Table S1E for data

Clustering at lower similarity thresholds decreases the total number of loci per sample (Figure 4c, Figure 5, Table S1E, F and G) and increases coverage depth per cluster (Figure 5), but the increase in coverage depth per cluster was minimal from 85% to 70% (i.e., <1 read per cluster). Clustering threshold values can, however, significantly affect the number of shared loci among lineages (Table S1H).

**Figure 6:**
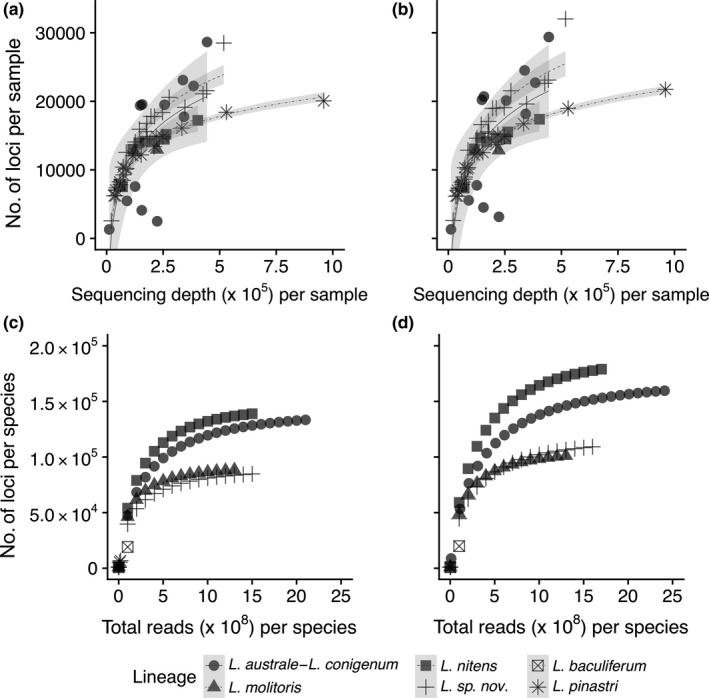
Reads vs. number of loci at 70% clustering (a & c) or 85% clustering (b & d). (a & b) Effect of sequencing depth (number of merged reads) per sample on the number of loci (*f2loci*) per sample. Fitted logarithmic curves are displayed for each putative species to represent accumulation curves. Gray regions indicate 95% confidence intervals of fitted curves. (c & d) The accumulation of new loci per species (unique *f2loci* within putative species) with increasing sequences. There were no enough samples or reads for *L. baculiferum* or *L. pinastri* for comparison

The relationship between sequencing depth and the number of loci recovered from each sample depended on the *Lophodermium* lineage (Figure 6). For example, whereas the loci accumulation curve for *Lophodermium sp. nov*. appeared to be reaching a plateau with greater sequencing depths, other species appeared to have a higher diversity of reads per sample. To test how sequencing depth differently affected the accumulation of new loci for different *Lophodermium* lineages, we merged shared loci per lineage and analyzed the loci accumulation curve using random sampling of loci with replacement. *L. nitens* and the *L. australe*–*L. conigenum* complex had significantly greater diversity of loci than *L. sp. nov*. or *L. molitoris* (Figure 6c, d). See Table S1F, G for details.

The average sequence length per locus decreased (i.e., from 262 to 249 bps) as singletons, potential paralogs, and loci with high rates of ambiguous nucleotides were excluded through the bioinformatic pipeline. The average sequence length of loci incorporated in the phylogenetic analyses (i.e., being shared with at least ten samples), however, did not significantly decrease (i.e., from 249 to 236 bps for 70/30N and from 246 to 242 for 85/15N).

### 3.3 Phylogenetic analyses

**Figure 7:**
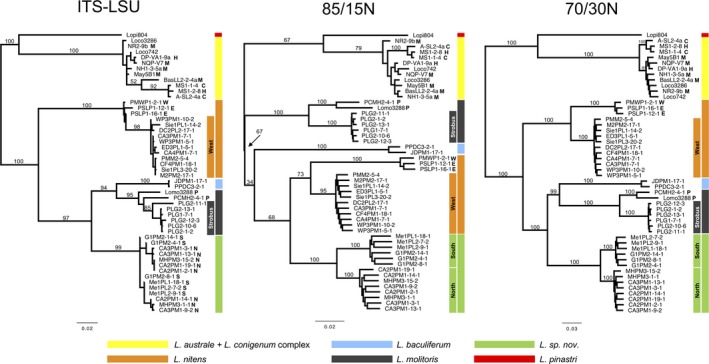
Unrooted maximum‐likelihood trees for nrDNA ITS‐LSU region and ddRAD loci obtained with two different combinations of similarity thresholds and number of allowed Ns in a read (see text for details). Bars next to the trees map isolates that belong to the same putative species or species complex. *L. australe* and *L. conigenum* are labeled after their original identifiers although they likely represent a single species and are referred as the *L. australe–L. conigenum* complex throughout this manuscript. *L. australe* isolates are labeled with M (major), C (cryptic), or H (hybrid) based on a population structure analysis by Oono et al. (2014). Putative *L. molitoris* isolates are labeled with P (*Pinus*) or shaded bars labeled *Strobus* based on host species. Putative *L. nitens* isolates are labeled E (East) or W (West) based on geographic origins. Putative *L. sp. nov*. isolates are labeled N (North) or S (South) based on geographic origins. Numbers above branches represent bootstrap values, not all bootstrap values are shown for a clearer view. See Figure S2 for ITS‐LSU phylogeny with additional reference sequences

The supported phylogenetic relationships, as revealed by ITS‐LSU alone, of the six putative species used in this study (Figure 7) were identical to those obtained with 55 additional reference *Lophodermium* sequences from public databases (Figure S2). The deeper branches had lower support (bootstrap values <75%) based on the 85/15N alignment compared to either the ITS‐LSU or 70/30N alignments (Figure 7). The 70/30N phylogeny had the highest bootstrap support in almost all branches (i.e., 100% bootstrap support), including branches supporting putative species. The tree topology recovered by the 70/30N matrix was also highly similar to the original ITS‐LSU tree. The main difference between the two topologies was that the 70/30N tree was better resolved in the terminal branches. The better‐supported branches within putative species often revealed interesting correlations between genetic structure and endophyte ecology. The new *Lophodermium* species and *L. nitens* both clearly consisted of two lineages, which mostly correlated with geography (i.e., north *vs*. south for *L. sp. nov*. and east vs. west for *L. nitens*; see Table S1A for details on geographic origins of samples). *L. molitoris* also consisted of two lineages, which correlated to different *Pinus* host subgenera (i.e., *Strobus* vs. *Pinus*). The two lineages of *L. australe–conigenum* complex could also be clearly delineated with the 70/30N dataset, but did not correlate with any known ecological differences.

The summary of final alignment matrices for 85/15N and 70/30N ddRAD loci is found in Table 1. The 85/15N matrix was not an inclusive subset of the 70/30N matrix, but contained 36 loci that were not found in the 70/30N matrix. We found that these 36 loci were discarded from the 70/30N matrix during the alignment stage because they either contained more than 100 SNPs across samples (11/36 loci) or more than 99 indels across samples (15/36 loci). The remaining loci (10/36) were excluded as potential paralogs because multiple haplotypes were found within a cluster. However, even when these 36 loci were excluded from the 85/15N matrix such that the 70/30N matrix was inclusive of all loci in the 85/15N matrix, the bootstrap support values did not significantly improve for the 85/15N matrix (data not shown).

**Table 1:** Summary of the three alignments used for phylogenetic analyses

Alignment	No. of Loci	Sum length of all loci	Polymorphic positions^a^	Ambiguous/Gap sites^b^ (%)	Missing loci^c^ (%)	Mean polymorphic positions per locus
ITS‐LSU	1	1,143	303	24.68	0.00	303
85/15N	2,068	529,469	54,335	77.78	76.99	26
70/30N	2,388	607,736	84,747	77.59	76.35	35

a Polymorphic positions only include nucleotide variants.

b Ambiguous/gap sites are either “N”s or “‐”s in the alignment matrix.

c Missing loci was calculated as the proportion of absent loci over the total number of potential loci (i.e., the number of samples multiplied by the total number of loci in a dataset).

**Figure 8:**
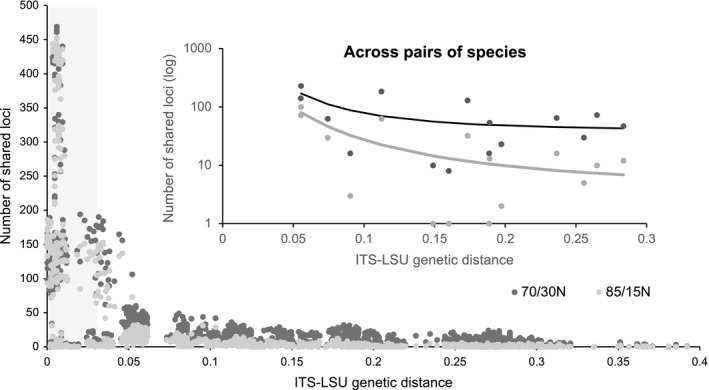
Number of loci clustered at 70% or 85% similarity with at least ten samples per locus that were shared between pairs of samples as a function of genetic distance, based on nrDNA ITS‐LSU sequences. The shaded area represents within‐species variation (>97% similarity). Inset: same data but merged to compare between pairs of putative species using a minimum of 2 species per locus. The two regressions represent fitted curves for each dataset, 70/30N (black) and 85/15N (gray). The equation for the fitted curves is *y* = *a* + *b*/*x* ^2^, where *a* = 37.842, *b* = 0.404, and *r* ^2^ = .404 for 70/30N matrix and *a* = 3.949, *b* = 0.234, and *r* ^2^ = .675 for 85/15N matrix

The number of loci shared within‐species was greater in the 70/30N criteria than in the 85/15N criteria (15.71% more loci, on average; Table S1F, G). The number of shared loci between sample pairs and species pairs decreases as genetic distances based on ITS‐LSU sequences increase (Figure 8). The decay rate of the number of shared loci with genetic distance is greater for the 85/15N matrix than for the 70/30N matrix. This pattern does not change when the number of shared loci is divided by the average number of total loci for pairwise comparisons (Figure S3).

### 3.4 Reproducibility

**Figure 9:**
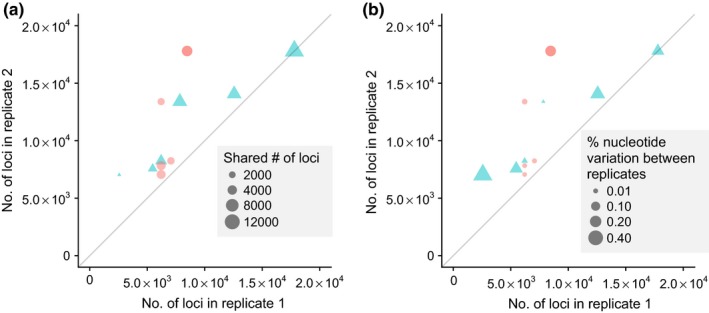
Effect of sequencing depth (total number of merged reads per sample) on (a) recovery of same (shared) loci (*f2loci*) and (b) nucleotide variation (observed error rate) between replicate pair samples filtered with the 70/30N criteria. Replicate samples run on same sequencing library are circles and different libraries are triangles. Markers are transparent, showing some apparent overlap in data values where they appear darker

The number of shared loci between technical replicate pairs varied between 1,254 and 12,340, most likely correlated to their ranges in sequencing depths (Figure 9a; Figure S4). The variation in base calls between shared loci of technical replicate pairs (observed error rates) ranged from 0.006% to 0.43% for the 70/30N dataset and 0.004% to 0.33% for the 85/15N dataset (Figure 9b, Figure S4, Table S1D). The observed error rates between replicates were significantly greater for 70/30N than for 85/15N analyses (paired *t* test, *p < *.0001), albeit a minor difference (0.0012 vs. 0.0007). The average estimated error rates were 0.22% and 0.24% for 70/30N and 85/15N datasets (Table S1F, G), respectively, based on the maximum‐likelihood equation of Lynch (2008). The average observed error rate was lower than the estimated error rate for both datasets (*p < *.05 for 70/30N and *p *<* *.01 for 85/15N). The estimated error rates did not significantly correlate with sequencing depths for the 59 samples (data not shown), but may have differed among *Lophodermium* lineages (Figure 6). Replicate pairs of the *L. sp. nov*. had lower error rates overall with an average of 0.01% (*n* = 6 pairs, 2 replicates) whereas pairs of *L. nitens* had an average of 0.24% error rate (*n* = 5 pairs, 3 replicates), but there were not enough replicate pairs for each lineage to test this statistically. The error rates between same and different sequencing libraries for three replicate samples were compared with a paired one‐way *t* test and were not statistically significant (*p *=* *.11) although there was a tendency for error rates to be higher between libraries than within (Table S1D).

## 4 DISCUSSION

The low‐cost ddRADseq, which had been previously applied for SNP variant discovery in a single species (Kess et al., 2016), generated a sufficient number of homologous loci to construct a strongly resolved phylogeny for multiple putative species of the widespread *Lophodermium* genus. We also found that genetic structure within putative species can often be correlated with geography or different host species, but it can also be observed within the same hosts and locations, suggesting that ecological traits other than dispersal limitation or host specificity can act as barriers to genetic introgression. The phylogenetic resolution was improved over ITS alone, but depended on filtering and clustering parameters. The clustering parameter was markedly more important than the filtering parameter.

### 4.1 Filtering and clustering criteria

Increasing the allowed number of Ns from 0 to 30 increased the proportion of filter‐passed reads, with the greatest increase between 0 and 5 Ns (Figure 3). The number of Ns was also positively correlated with number of clusters (including singletons), coverage depth (Figure 4a), and number of loci (excluding singletons; Figure 4c), but the increases in the latter two were modest after 5 Ns. Applying a less‐stringent filtering criteria only led to removal of greater proportions of clusters later in the pipeline (Figure 4b) because more clusters either were singletons, had consensus sequences with greater than five Ns, or were potential paralogs (i.e., had more ambiguous nucleotide positions within clusters). Overall, the number of allowed Ns had little effect on the number of shared loci among our *Lophodermium* species that were used in the phylogenetic analyses (Figure 4d). This suggests that allowing a reasonable number of Ns does not bias the final result of this study but may marginally increase the average coverage depths for more accurate consensus sequences and fewer potential paralogs to be excluded due to ambiguous sites in reads.

Increasing clustering thresholds from 70% to 85% or 95% increases number of loci per sample (Figure 6) but decreases the number of shared loci among different *Lophodermium* lineages (Figure 8, Table S1H). Interestingly, although we typically consider deeper coverage to be associated with decreased error rates, lower clustering thresholds had greater error rates (Table S1D) despite, albeit marginally, deeper coverage (Figure 5). The error rates were, however, both minor. Therefore, we suggest various clustering thresholds to be tested that minimizes error rates (i.e., compare replicates) and maximizes shared loci at the genetic scale and breadth of each study.

### 4.2 Phylogenetic topologies with ddRAD loci

The phylogenetic reconstruction based on the 70/30N matrix was significantly better resolved in the deeper branches than by the 85/15N matrix and also in the terminal branches than by the ITS‐LSU matrix (Figure 7). The well‐resolved terminal branches of the 70/30N tree corresponded to previously identified (e.g., *L. australe* major *vs*. cryptic; Oono et al., 2014) or potential cryptic species (within *L. nitens* and *L. sp. nov*.). For instance, the two well‐supported clades within *L. sp. nov*. (Figure 7, Figure S2) correspond to samples recovered from two distinct geographical regions, Northern California and Oregon/Washington, which may correspond to two species or a single highly structured species. The 70% threshold allowed the inclusion of highly variable loci, which increased the number of phylogenetically informative positions and shared loci between more distantly related species (Table 1, Figure 8, Table S1H). Although clustering reads or loci by similarity is an imperfect solution to identifying homologous loci, our results agree with conclusions from Rubin et al. (2012), showing that clusters that may include a mixture of orthologous and paralogous loci still contain substantial phylogenetic signal that can produce correct topologies with moderate to high accuracy.

The phylogenetic reconstruction based on the 85/15N matrix still improved over that of the ITS‐LSU matrix for within‐species structure (i.e., recent divergence) despite lower bootstrap values at deeper branches. A higher clustering threshold of 85% significantly diminished the number of shared loci across species compared to 70%. For instance, while *L. nitens* and the *L. australe*–*L. conigenum* complex (the two most distantly related species in our sample) had only ten shared loci in the 85/15N matrix, there were 73 in the 70/30N matrix (Table S1H). This sevenfold difference contrasts with the closely related species *L*. *baculiferum* and *L. molitoris*, whose number of shared loci in 85/15N and 70/30N was different by twofold (30 and 63 loci, respectively). This result is consistent with the observation that the number of shared loci decreases abruptly with genetic distance for both thresholds but is more pronounced for 85/15N (Figure 8). As a result of using a different number of allowed Ns and clustering thresholds, the 85/15N matrix included 36 loci that were not found in the 70/30N matrix. These loci, however, were not solely responsible for the lower support of deeper branches by the 85/15N matrix. As explained before, it is more likely that this matrix performed worse because of fewer shared loci across distantly related species.

The number of homologous loci in distantly related lineages can likely be improved with greater sequencing depths or narrower selection of sequence lengths. In this study, as the genetic distance between species increased, the number of shared loci decreased, although not at a linear rate. Hence, this ddRADseq method is especially effective in revealing within‐species fungal (<97% ITS similarity) diversity, but is also cost‐effective for among‐species (e.g., 97–70% ITS similarity) diversity. However, restriction site polymorphism will likely limit the efficacy of ddRADseq for phylogenetic reconstruction beyond the genus and family levels (DaCosta & Sorenson, 2016). Furthermore, the mutation risk hypothesis suggests that fewer homologous or shared loci will be recovered for loci with greater risk of mutations. Hence, longer MiSeq ddRAD reads, which have greater likelihood of mutations that create novel restriction sites within reads, may have been less likely to be shared among species than within species (Arnold, Corbett‐Detig, Hartl, & Bomblies, 2013; Gautier et al., 2013). Simple mutations in restriction sites may also increase the read length variation for longer MiSeq reads compared to shorter HiSeq reads, but we saw some sequences with restriction sites incorporated in the middle of the sequence, suggesting that length variation can also be caused by inefficient enzyme digestions. Length variation caused by inefficient enzyme digestions or mutations at restriction sites may cause lower rates of clustering, but should not bias phylogenetic analyses because gaps are treated as missing data. Trimming reads to shorter lengths or targeting shorter reads will likely minimize loss of clusters or loci due to length variation among and within samples of different species. However, using longer reads produces more informative sites that may compensate for having a reduced number of shared loci and can potentially resolve deeper divergences (e.g., >300 million years) better than shorter reads (Rubin et al., 2012). Longer reads can also be more helpful for downstream population genetic analyses, such as genome mapping, haplotype reconstruction, and linkage to adaptive loci. Our case study shows that small portions of the genome sequenced with the MiSeq platform can be sufficient to produce comparative homologous loci within highly diverse taxa with small haploid genomes (Figures 6 and 7, Table S1H). Other ddRADseq datasets that are not based on strict nucleotide polymorphisms, such as indel or presence–absence polymorphisms, may further help the resolution of more distantly related lineages (DaCosta & Sorenson, 2016).

### 4.3 Reproducibility analysis

The observed error rates between replicate samples that were run within or between libraries were low, averaging about one error every 1000 base pairs (e.g., 0.0012 for 70/30N criteria, Table S1D) and lower than expected error rates. The observed error rates were also not significantly different when samples were run in the same or different libraries, but the error rates tended to be higher between libraries than within (Table S1D). Replicate samples in different libraries represent different periods of laboratory preparation (i.e., PCR group, enzyme efficiency) which may present variation and increasing error rates than between replicate samples in the same library. Furthermore, the marginal increase in coverage depth and decrease in error rate with increasing sequencing depth may have depended on the *Lophodermium* species if they had different genome sizes.

Increasing sequencing depth per sample is likely the best method to improve reproducibility. Sequencing depths correlate with coverage depth per cluster (Figure S1), which affects the estimation of nucleotide identity when there are discrepancies among reads within a cluster. Hence, increasing sequencing depth increases reproducibility among samples and between libraries by more accurately distinguishing PCR or sequencing errors from polymorphisms with greater coverage (Figure 9). Sequencing depth also has a strong positive correlation with number of loci per sample (Figure 6) and therefore increases the probability of sequencing shared loci among samples. Narrowing the range of read lengths to be sequenced during preparation of the pooled library would also help improve coverage. However, as we saw, deeper sequencing or greater coverage per locus (i.e., lower error rates within locus) was not necessarily needed for improving phylogenetic assessments within this genus. Clustering at a lower threshold had the greatest effect on improving phylogenetic resolution by identifying more shared loci across distantly related species. A fine‐scale analysis for population structure and diversity may require better resolution of nucleotide variation within clusters with greater coverage depths. For the identification of heterozygosity in nonhaploid organisms or homologous loci of larger genomes (e.g., >100 Mbps), deeper sequencing depths will be necessary with additional modifications, such as adapters that include random degenerate sites for identifying PCR duplicates (Hoffberg et al., 2016), a narrower selection of read lengths, multiplexing fewer samples per library, or applying this protocol on the HiSeq. This study suggests that for haploid fungi that have relatively small genome sizes (30‐50 Mbps; Tavares et al., 2014; Gregory et al., 2007), increasing sequencing depths beyond 200k per sample will sufficiently decrease the error rates per locus and will be robust for fine‐scale genetic analyses.

## 5 CONCLUSIONS

The low‐cost ddRADseq protocol using standard indexes (Kess et al., 2016) produced sufficient numbers of loci to resolve the phylogenetic relationships of a diverse genus of fungal endophytes at lower costs (see Appendix S2 for cost comparison analysis) than the original ddRAD protocols. Special attention is needed, however, to identify appropriate filtering and clustering parameters. Although clustering thresholds significantly affected the phylogenetic resolution, quality‐filtering had little impact. Reproducibility and coverage depths were linked to sequencing depths, but high coverage depths (e.g., >10 typically found in HiSeq data analyses) were not essential for strong phylogenetic support in this taxonomic group with a relatively small haploid genome. The use of longer reads may have reduced the number of shared loci, but the marginal increase in phylogenetically informative sites per read may compensate for this disadvantage.

## CONFLICT OF INTEREST

None Declared.

## AUTHORS’ CONTRIBUTIONS

RS‐L and RO conceived the ideas and designed methodology; RS‐L and RO collected the data; RS‐L and RO analyzed the data; RS‐L and RO wrote the manuscript. All authors contributed critically to the drafts and gave final approval for publication.

## DATA ACCESSIBILITY

Data and R codes used for Figure 6 are available at DataOne https://doi.org/10.15146/r31m36. Phylogenetic trees and alignment files are available at TreeBASE S22555.
