# Supplementary material for: Double‐digest RADseq loci using standard Illumina indexes improve deep and shallow phylogenetic resolution of Lophodermium, a widespread fungal endophyte of pine needles
Source: Ecol Evol. 2018 Jun 11;8(13):6638–51. doi: 10.1002/ece3.4147 (PMC6053583; doi:10.1002/ece3.4147)
Supplement: Supplementary file 1 [file ECE3-8-6638-s001.pdf]

Supporting Information Figures

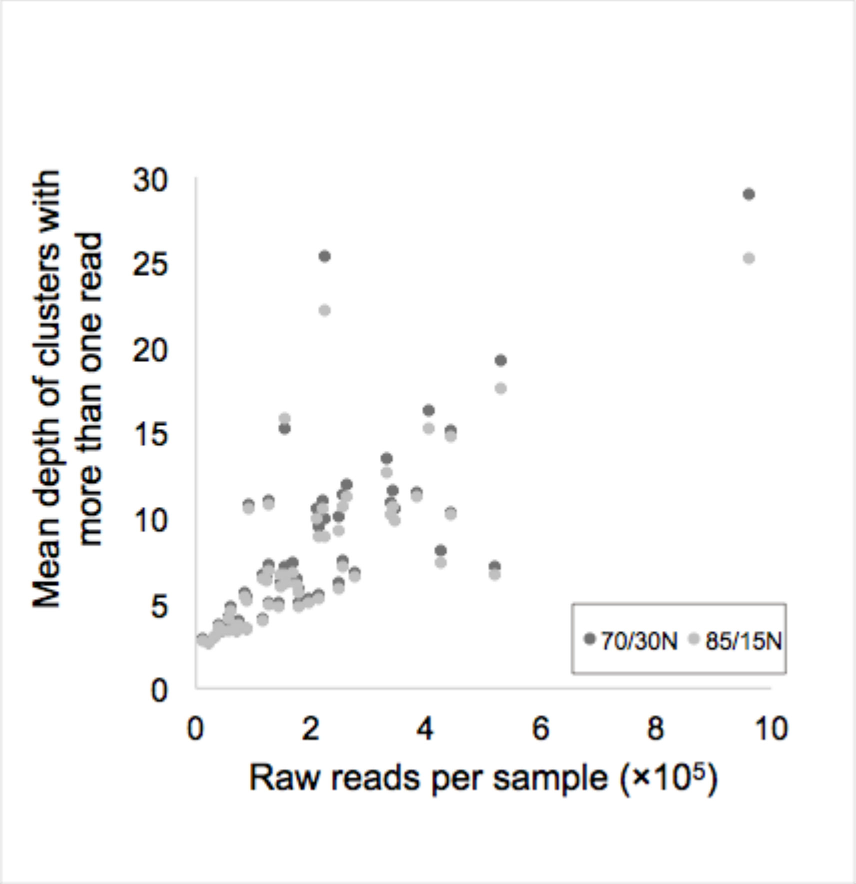

Figure S1. The mean depths per cluster, excluding singletons, as a function of sequencing depth per sample (Spearman's rank correlation, p < 0.001 for both 70/30N and 85/15N datasets).

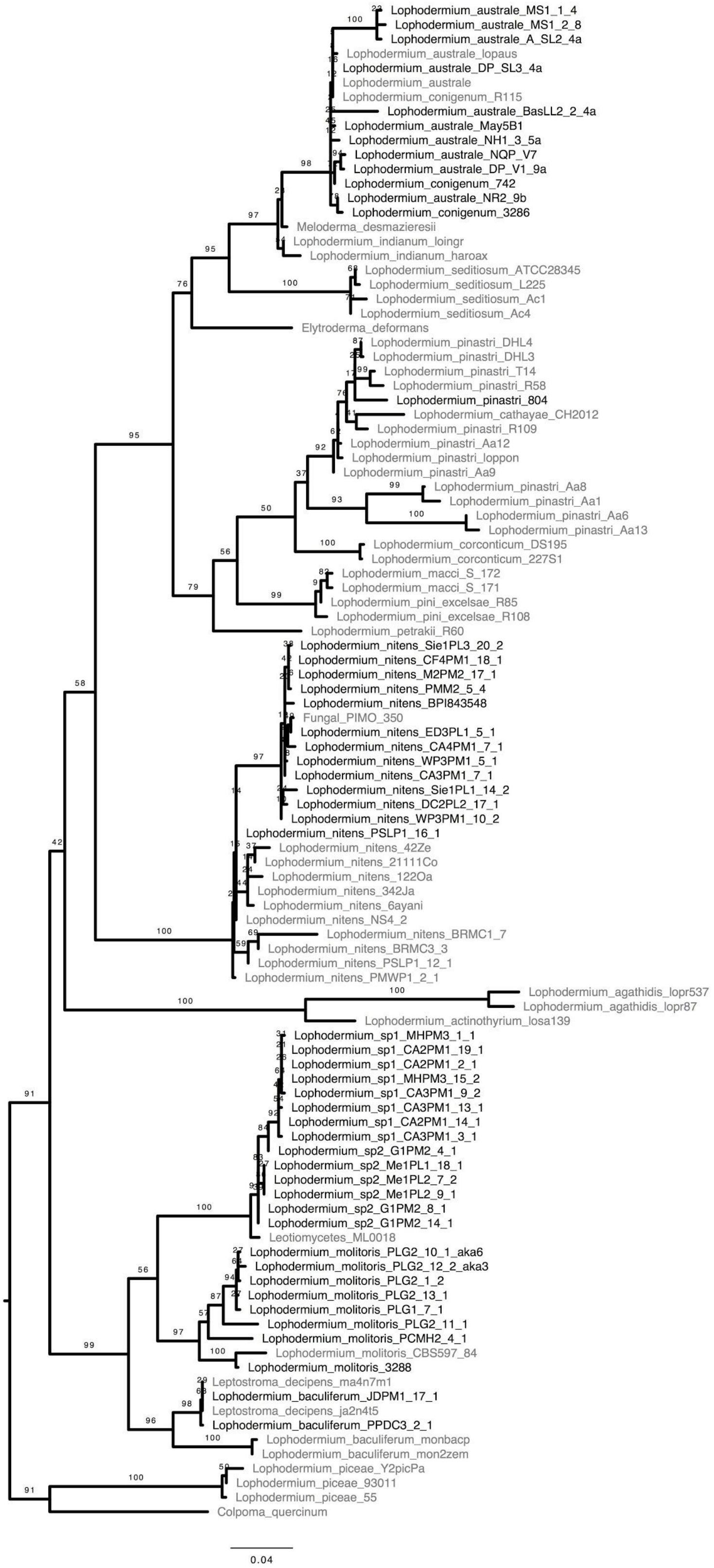

Figure S2. Maximum Likelihood tree of ITS-LSU for the 50 *Lophodermium* samples (black labels) used in this study along with 55 reference sequences (grey labels). Numbers above branches are bootstrap values with 1000 bootstrap replicates. See Table A for GenBank accession and metadata information of the isolates sampled in this study and Table B for GenBank and UNITE accessions of reference sequences.

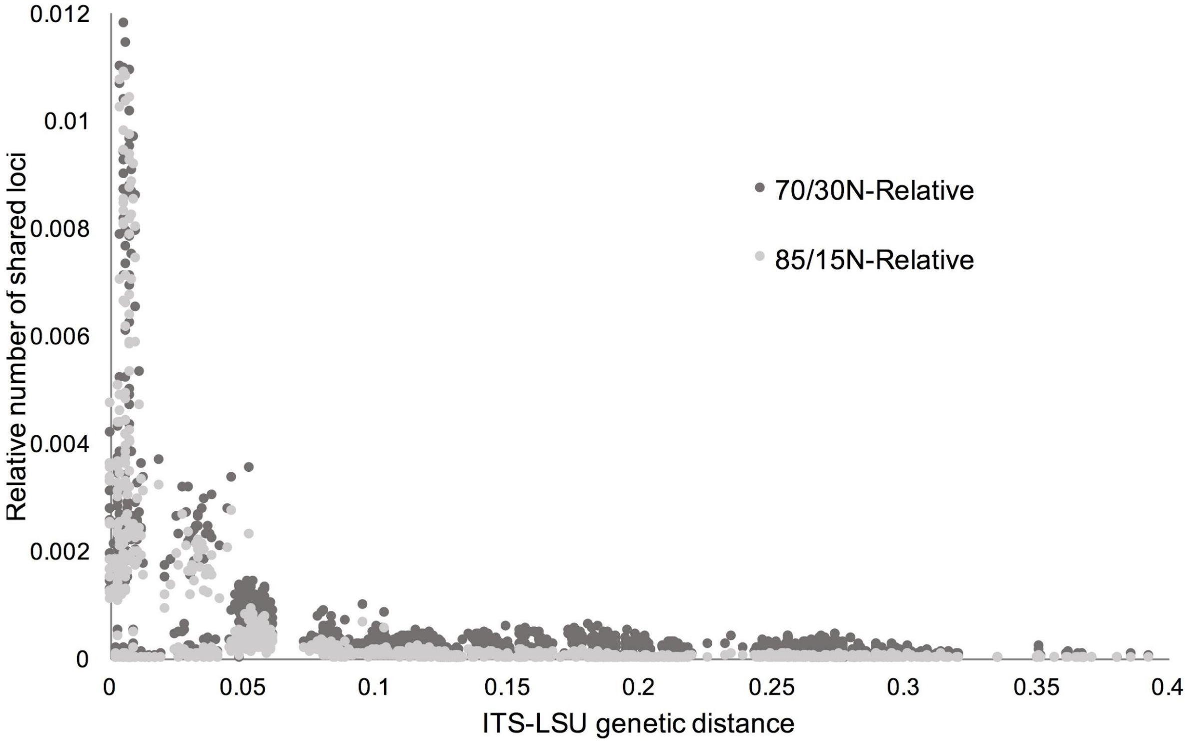

Figure S3. Relative number of loci clustered at 70% or 85% similarity with at least ten samples per locus that were shared between pairs of samples as a function of genetic distance, based on nrDNA ITS-LSU sequences. For each pairwise comparison, the relative number of shared loci was calculated by dividing the total number of shared loci (data in Fig. 7) by the average number of loci of each compared pair (*nloci* columns in Table F & G) after quality filtering.

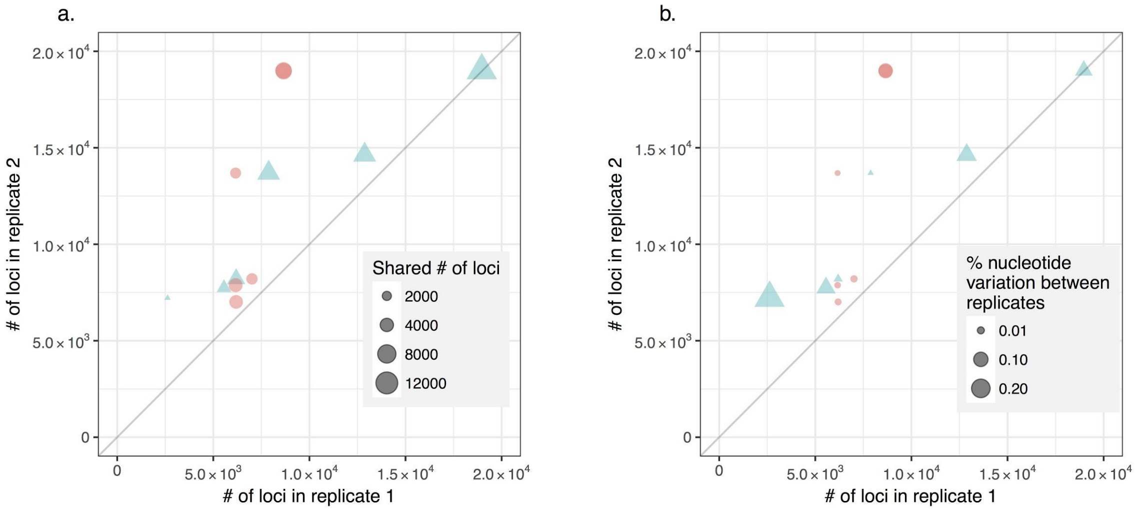

Figure S4. Effect of sequencing depth (total number of merged reads per replicate sample) on a) recovery of same (shared) loci (*f2loci*) and b) nucleotide variation (observed error rate) between replicate pair samples filtered with the 85/15N criteria. Replicate samples run on same sequencing library are circles and different libraries are triangles.
