# Supplementary material for: Double‐digest RADseq loci using standard Illumina indexes improve deep and shallow phylogenetic resolution of Lophodermium, a widespread fungal endophyte of pine needles
Source: Ecol Evol. 2018 Jun 11;8(13):6638–51. doi: 10.1002/ece3.4147 (PMC6053583; doi:10.1002/ece3.4147)
Supplement: Supplementary file 3 [file ECE3-8-6638-s003.pdf]

Appendix 1: (not so) **Short CTAB DNA Extraction Protocol**
Modified from Sara Branco's Protocol
http://1000.fungalgenomes.org/home/protocols/high-quality-genomic-dna-extraction/

This protocol was used successfully to extract high quality DNA (up to 600 ng/µL) of *Lophodermium* spp. Downstream applications include ddRAD-seq and whole genome sequencing.

[Comments and notes within brackets]

Before starting...

- Turn on the bath and set it to 65ºC.
- While waiting for the bath to warm up, label all microcentrifuge tubes.

If using fungal mycelium from liquid culture:

- Recover mycelia from liquid by decanting the media; then use filter paper to dry mycelia as much as possible. After having carefully squeezed the mycelium with the filter paper, dry the tissue over the filter paper for 15-20 min in sterile laminar-flow hood.
  [I usually collect the mycelia in two separate tubes, one for CTAB and one for backup at -80ºC freezer]
- **Critical step.** Grind tissue (0.04-0.2g, more tissue is not recommended) to a very fine powder using liquid N_2 with mortar and pestle
  [If low DNA yields were obtaining from the starting tissue, try more than one extracion, combine the extracted DNA and reduce volume by precipitating DNA following steps 9 to 12 and resuspend as described in step 15. Freeze mortar and pestle with N_2 before start grinding. Try to grind completely the tissue with no more than a couple N_2 shots. Do not over-freeze the sample or the CTAB buffer will freeze when added and it will take a long time before it melts again.]

Protocol (Takes about 5 hours long, can be paused at **step 9** if necessary.)
1. Add 800µL of 2X CTAB buffer (see recipe below) per sample to the 0.2-0.5 g of ground mycelium in the mortar and keep grinding (if it is frozen, then wait until it is melted again, but do not leave sample melted for long period).
   [2X CTAB buffer and 2% PVP-40 can be mixed in advance and stored at room temperature.
   Volume is for each tube, i.e. if using 2 tubes per sample, then add 2×800µL = 1.6mL]
2. Recover CTAB + ground mycelia (approx. 750 µL per 2mL microcentrifuge tube).
   [If necessary, this solution can be stored in the fridge for up to one week for later extraction. If so, wait until the tubes reach room temperature before starting next step.]
3. **Use the fume hood, wear coat and double gloves**. Add 2.5µl β-mercaptoethanol to each tube.
   [I always pipet a small amount of β-mercaptoethanol in a 600µL microcentrifuge tube to avoid repeat-pipetting in the original bottle.]
4. Incubate at 65ºC for 30 minutes, shake samples every 10 minutes.
5. Add 1X volume of chloroform isoamyl alcohol (24:1), shake for 20 min (100-150 rpm), position tubes horizontally for better mixing.
   [If possible, move the shaker into the fume hood.]
6. Spin down at max speed for 5 minutes.
7. Transfer the top phase to a 1.5 mL fresh tube and add 5 µL of RNAse stock solution (1 µg/µL) for each 100µL of recovered volume, incubate 30min at 37ºC.
   [I normally recover 400-450µL, so I add 20-22 µL of RNAse.]
8. Repeat **step 5** but only shake each tube briefly this time. Repeat **step 6**, then skip to **step 9**.
9. Transfer the top phase to a 1.5 mL fresh tube. Then add 15µl 5M potassium acetate and ⅔X volume of **room temperature** isopropanol to each tube. Mix the sample gently by inverting.

[Ice-cold isopropanol enhances precipitation of DNA but may also cause some DNA degradation. I use room-temperature isopropanol and then a 30 min incubation at -20ºC. If necessary, protocol can be paused here overnight.]

10. Spin down 3 min at max speed. Discard isopropanol by decantation or with a pipette without touching the pellet.

11. Wash the pellet with 750µl **ice-cold** 70% ethanol, making sure the pellet is loose and has been washed throughout. Incubate at room temperature for at least 5 min.

12. Spin down for 1 min and remove ethanol by decantation or with a pipette, do not touch the pellet.

13. Repeat **steps 11** & **12**.

[I use 70% ethanol for both washes.]

14. Spin down for 1 minute. This time, removing ethanol with a pipette is preferred over decantation. Do not touch the pellet. Dry in hood for about 15 min. Do not overdry the pellet (or it would be very hard to resuspend).

[Pre-warm TE at 65ºC.]

15. Reconstitute with pre-warmed 30-50µl TE buffer**.** If necessary (if the pellet does not dissolve immediately), incubate samples at 65ºC for about 15 min.

[I usually wait for the next day before running a gel (**Figure 1**) or quantifying concentration to allow all DNA to dissolve completely.]

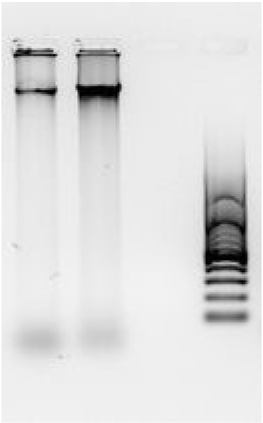

Appendix 1 Figure 1 Electrophoresis 1.5% agarose gel after running in 1X TAE buffer 100V for 40 min. The first two lanes (left to right) are two samples extracted with this protocol, the third lane is empty, the fourth is a 100 bp ladder.

2X CTAB Buffer (add ingredients in the order of the list):

100 mM Tris-HCl (pH 8.0)
1.4 M NaCl
20 mM EDTA
2% CTAB
2% PVP-40
