# Supplementary material for: Double‐digest RADseq loci using standard Illumina indexes improve deep and shallow phylogenetic resolution of Lophodermium, a widespread fungal endophyte of pine needles
Source: Ecol Evol. 2018 Jun 11;8(13):6638–51. doi: 10.1002/ece3.4147 (PMC6053583; doi:10.1002/ece3.4147)
Supplement: Supplementary file 4 [file ECE3-8-6638-s004.docx]

**Appendix 2: Protocol for double digest RAD (ddRAD) sequencing with standard Illumina indexes**

This protocol was originally modified from Mastretta-Yanes *et al.* [(2015)](https://paperpile.com/c/3t2TZZ/9lHy/?noauthor=1) and Wyss *et al.* [(2016)](https://paperpile.com/c/3t2TZZ/58wg/?noauthor=1) but represents a variant of the protocol by Kess *et al*. (2016). The definitions of ‘barcode’, ‘index’, ‘library’ and ‘fragment’ are based on the original ddRAD protocol [(Peterson et al. 2012)](https://paperpile.com/c/3t2TZZ/bLzL).

**SUMMARY OF MODIFICATIONS:**

- Ligation adapters match EcoRI and MseI enzymes (see [**Adapters sequences**](#lcyd86h87eqe) at end).
- Adaptors were designed to match Nextera Transposase Adapters for single or paired-end DNA sequencing and Nextera Index kit v2 (Illumina Document #1000000002694 v03 October 2017, page 12).^^[[1]](#footnote-1)^^
- Multiplexing relies on PCR indexed primers as in Kess *et al.* (2016) and not, as in other ddRAD protocols, on the combination of barcoded adapters and indexed PCR primers. In other words, a unique combination of indexes is added to each individual sample during PCR amplification (See **step 4** below).
- Illumina Nextera sequencing primers (R1 & R2) were modified, as in Kess *et al.* (2016), to incorporate the restriction sites of the targets (see **Modified** [**Illumina sequencing primers**](#wxawu1eqqbfy) at end).
- [Cost comparison](#m6axzwptn36j) with Peterson et al. (2012) with 96 samples at end.

**Future potential modifications**: The present design of adapters and indexed primers does not include any strategy to detect PCR duplicates. Recommendations of Hoffberg et al. [(2016)](https://paperpile.com/c/PIaoFk/U5ZB/?noauthor=1), i.e. incorporating a number of degenerate sites to adapters, could be added and tested in the future.

**PREPARATION BEFORE DIGESTION:**

**Starting DNA material** (see [Appendix 1](http://tinyurl.com/zdlavfr) for details) should be at least 50 ng/μL. If concentration is below that, double digestion and double ligation reactions could be performed on multiple replicates and then pooled after ligation, as long as dilution in step **2d** is made according to a final volume of 40 μL.

**Adapters should be annealed** prior to starting the digestion protocol. Each adapter is a double stranded DNA composed of two oligos ([see **Adapter sequences**](#lcyd86h87eqe) at end). These adapters lack barcodes. Therefore, all P1 and P2 adapters are identical and only each complementary pair should be annealed separately as follows:

- **Adapter P1:** Mix **EcoRI-P1.1** and **EcoRI-P1.2** oligos to a final concentration of 1μM of each oligo.
- **Adapter P2:** Mix **MseI-P2.1** and **MseI-P2.2** oligos to a final concentration of 10μM of each oligo.

Anneal oligos in a thermal cycler; 95°C 5 min, followed by a 0.1°C /seg ramp down to 20°C. Keep small aliquots of annealed adapters in the freezer.

**Randomization of samples in a plate is advisable in order to avoid bias.**

**PROTOCOL:**

**Step 1. Double restriction digest**

This step produces genome fragments with either one or two different ends (one for each enzyme).

**1a** Prepare master mix, vortex, and centrifuge. Make calculations for 1.2 X # of samples. Include a negative control.

dd master mix EcoRI + MseI Vol (μL)

10X T4 DNA Ligase Buffer 0.9

1 M NaCl 0.45

1 mg/mL BSA 0.45

NF water 1.00

MseI (10,000 U/mL) 0.10

EcoRI (100,000 U/mL) 0.10

Total mix volume per sample 3.0

*Note:The T4 DNA Ligase Buffer should be thawed and resuspended at room temperature.*

**1b** Mix 3 μL of dd master mix with 6 μL of DNA in each PCR tube or well.
*Note: Pipette DNA carefully to avoid cross contamination.*

**1c** Centrifuge briefly and incubate at 37°C for 4 hours on a thermal cycler with a heated lid. Heat kill the enzymes at 65°C for 10 min. Keep samples at 4°C.

**Optional:** Use Tape Station or any other Fragment analyzer to confirm digestion and evaluate size and density of fragments (see Peterson protocol). Perform double and single digestion reactions of each enzyme for a small sample subset. Use dd master mix above replacing NF water for each enzyme in single digestions ([**Figure 1**](#i9g2dl84ypen)).

*Note:* It is very advisable to perform this step when conducting pilot experiments and/or if no additional information about the genome of the studied species is available (see Peterson *et al.* [2012] protocol and links therein for more information).

**Step 2. Ligation**

This step ligates P1 and P2 adapters to digested fragments.

**2a** Prepare Ligation master mix as follows:

Ligation Master Mix EcoRI + MseI Vol (μL)

10X T4 DNA Ligase Buffer 0.26

1 M NaCl 0.13

1 mg/mL BSA 0.13

NF water 0.0125

P1 (EcoRI) adapter 1 μM 1.0

P2 (MseI) adapter 10 μM 1.0

T4 DNA ligase (400,000 U/mL) 0.1675

Total mix volume per sample 2.7

*Note:The T4 DNA Ligase Buffer should be thawed and resuspended at room temperature.*

**2b** Add 2.7 μL of ligation mix to each PCR tube or well of digested DNA. Total volume becomes 11.7 μL. Mix, centrifuge and incubate at room temperature (21°C approx.), overnight.

**2c** Heat kill enzyme at 65°C for 10 min.

**2d** Dilute ligation reactions to a volume of 40 μL using Tris 10 mM

*Note: Remember to pool replicate double digestion-ligation reactions before dilution (see note about starting DNA concentration).*

**Step 3. AMPure clean-up**

This step helps eliminate undesired small fragments, unligated fragments, and adapter dimers.

*Note: Working with no more than 48 samples at once gives best results.*

Clean the ligation product with AMPure XP beads. Use volume 1X to keep fragments above 200 bps or 0.8X for fragments above 300bps.
*Note: 0.8X recommended for MiSeq runs; since samples are 40 μL, 0.8X = 32 μL.*

**3a** Make fresh 80% ethanol. Make a total of 180μL x 2X # of samples. Label new PCR tubes or plate. High profile tubes work best.

**3b** Remove beads from the fridge and shake well. Transfer beads into smaller container for ease of allocation.

**3c** Add 0.8X volume of AMPure beads to each tube or well and mix by pipetting.

**3d** Incubate for 5 min. at room temp.

**3e** Place PCR tubes or plate on the magnet plate. Incubate for 2 min.

**3f** Pipette out supernatant without disturbing the beads.

**3g** Add 180 μL of 80% ethanol to each tube or well. Incubate for 30 s. Pipette out the ethanol.

**3h** Repeat wash from previous step. Remove all ethanol by pipetting.

**3i** Allow ethanol to evaporate in the fume hood for 5-10 min.

**3j** Remove PCR tubes or plate from magnetic plate and add 1X volume of 10 mM Tris buffer (i.e., 35μL). Mix well by pipetting up and down and incubate for 2 min.

**3k** Place samples on the magnetic plate for 1 min. Pipette out the supernatant into new PCR tubes.

*Note: See* [***Figure 2***](#szj8dtpyn) *for a comparison of double digested/ligated fragments before (step 2) and after (step 3) cleanup.*

**Step 4. PCR amplification**

This step incorporates Nextera Index Kit v2 primers with unique indexes to the adapters (which contain the dd fragments) and enrich the library before sequencing.

*Note: A unique combination of P5 and P7 indexed primers are used for each individual. Therefore it is advisable to prepare a plate (or tubes) with each pair of primers with anticipation for this step. Use fresh primer aliquots for better results. Include a negative control to measure background primer dimer concentration.*

**4a** Prepare the PCR master mix (total volume 25 μL). Include negative control.

PCR Master Mix Vol (μL)

NF water 9.725

1 mg/mL BSA 2.5

8mM dNTP 3.125

10X PCR (with MgCl_2_) 2.5

Taq 0.15

Total mix volume per sample 18

For each individual sample (DNA fragments cleaned from **step 3),** add a unique combination of P5 & P7 primers:

P5 primer 1 μL

P7 primer 1 μL

DNA 5 μL

**4b** Perform two separate PCR reactions for each sample by splitting all samples into two different tubes, 12.5 ul reaction in each.

**4c** PCR program. 98°C for 30 s; 14 cycles of 98°C for 20 s, 60°C for 30 s, 72°C for 40 s; final extension at 72°C for 10 min.

**4d** Combine separate PCR reactions in a single well (or tube) for each sample.

**4e** Confirm PCR reaction success in a 1.5% agarose gel ([**Figure 3**](#fcg9t31c10w4)).

**Step 5. Pooling library**

**5a** Quantify PCR concentration for all samples, including the negative control and (optional) verify the presence of a smear using a 1.5% gel, 65V for 1 hour. Use Qubit to quantify DNA also.
*Note: If some samples are low in concentration (lower than the concentration of the negative control ~3 ng/ul) but a faint smear is visible on the gel you may re-run those samples on a new PCR increasing one or two cycles.*

**5b** Pool all samples in equimolar proportion. If all the samples have approximately the same mean size, there is no need to convert to nM (i.e. use concentration in ng/uL to pool all samples).
*Note: Consult your sequencing provider about what are the ideal volume and final concentration of your library.*

**5c** Optional. Reconcentrate library. Add 1/10 vol KAc 3M (or any other salt acetate, like NaAc) and 2X 100% Ethanol and keep in the freezer for at least 30 min.

*Note: the* ***minimum*** *DNA initial concentration must be above 1 ng/μL taking into account KAc and Ethanol volumes; otherwise it would not precipitate and will be lost.*

**5d** Centrifuge at maximum speed for 15 min, remove supernatant carefully.

**5e** Add 200 μL 70% ethanol and centrifuge for 10 min, remove supernatant carefully.

**5f** Dry the pellet for 5 min in the fume hood and resuspend using 20-40 μL of Tris 10mM.
 *Note: Resuspend in an appropriate volume keeping in mind the limits of Pippin Prep cassettes used in the next step (30μL per lane) and the desired concentration of your library.*

**Step 6. Size selection**

This step is needed to keep fragments within a range of potential homologous sequences among individuals and eliminate all undesired small fragments, PCR primers, etc.

**6a** Quantify final library concentration using Qubit.

**6b** Use Pippin Prep Agarose cassettes and keep fragments of desired size.
*Note: The maximum volume for each cassette is 30 μL and maximum concentration is 10 μg of sheared genomic DNA. Chose the appropriate concentration of agarose cassettes given the size range of fragments to recover. We kept fragments between 300-700 bp for MiSeq using 1.5% agarose cassettes in five lanes.*

**6c** Pool size-selected products from Pippin Prep (if more than one lane was used) and quantify library concentration again.
*Note: Some DNA is lost during Pippin Prep.*

**6d** Convert ng/μL to nmol using nmol = (ng/μL/(660*500)*1000000); where ng/μL is the concentration and 500 is the mean size of the library. Verify that the library is above 5 nmol, **do not** dilute if it is above that.

**Optional**

Use Tape Station or any other Fragment analyzer with the resulting library after step **6c** to confirm that the desired size selection was made ([**Figure 4**](#lz2dmum2vxlh)).

**Step 7. Send library to the sequencing facility
7a.** Label 1.5mL tube and send >26 μL to the sequencing facility.
***Important note:*** Remember that Illumina sequencing primers for this library need to incorporate cutting sites for each fragment edge, otherwise sequencing will fail. See Modified Illumina Nextera [sequencing primers](#wxawu1eqqbfy) below.

**REFERENCES**

Hoffberg, S.L., T.J. Kieran, J.M. Catchen, A. Devault, B.C. Faircloth, R. Mauricio, and T.C. Glenn. 2016. “RADcap: sequence capture of dual-digest RADseq libraries with identifiable duplicates and reduced missing data.” *Molecular Ecology Resources* 16 (5): 1264-1278.

Kess, T., J. Gross, F. Harper, and E. G. Bouding. Low-cost ddRAD method of SNP discovery and genotyping applied to the periwinkle *Littorina saxatilis*. *Journal of Molluscan Studies* 8 (1): 104-109.

[Mastretta-Yanes, A., N. Arrigo, N. Alvarez, T. H. Jorgensen, D. Piñero, and B. C. Emerson. 2015. “Restriction Site-Associated DNA Sequencing, Genotyping Error Estimation and de Novo Assembly Optimization for Population Genetic Inference.” *Molecular Ecology Resources* 15 (1): 28–41.](http://paperpile.com/b/3t2TZZ/9lHy)

[Peterson, Brant K., Jesse N. Weber, Emily H. Kay, Heidi S. Fisher, and Hopi E. Hoekstra. 2012. “Double Digest RADseq: An Inexpensive Method for de Novo SNP Discovery and Genotyping in Model and Non-Model Species.” *PloS One* 7 (5): e37135.](http://paperpile.com/b/3t2TZZ/bLzL)

[Wyss, Tania, Frédéric G. Masclaux, Pawel Rosikiewicz, Marco Pagni, and Ian R. Sanders. 2016. “Population Genomics Reveals That within-Fungus Polymorphism Is Common and Maintained in Populations of the Mycorrhizal Fungus Rhizophagus Irregularis.” *The ISME Journal* 10 (10): 2514–26.](http://paperpile.com/b/3t2TZZ/58wg)

**FIGURES AND SEQUENCES**

**Adapter sequences:**

| MseI-P2.2 | /5Phos/**TA**CTGTCTCTTATACGAGAACAA | $75.81 for 100 nM |
| --- | --- | --- |
| MseI-P2.1 | GTCTCGTGGGCTCGGAGATGTGTATAAGAGACAG | $62.86 for 100 nM |

| EcoRI-P1.1 | GTCGGCAGCGTCAGATGTGTATAAGAGACAGC | $60.04 for 100 nM |
| --- | --- | --- |
| EcoRI-P1.2 | /5Phos/**AATTG**CTGTCTCTTATACACATCTGACGCTGCCGACGA | $82.85 for 100 nM |

/5Phos/ means Phosphorylate. All sequences are 5’-3’ oriented. Price for adapters are estimated from Integrated DNA Technologies from September 2017.

**Modified Illumina Nextera sequencing primers**:

| ddRAD R2 | GTC TCG TGG GCT CGG AGA TGT GTA TAA GAG ACA G**TA A** |
| --- | --- |
| ddRAD R1 | TCG TCG GCA GCG TCA GAT GTG TAT AAG AGA CAG **CAA TTC** |

All sequences are 5’-3’ oriented. Nucleotides in bold correspond to the restriction sites. In this case, primer ddRAD R1 contains EcoRI restriction site and primer ddRAD R2 includes MseI site. To incorporate a different pair of enzymes, simply replace the bolded sequences in ddRAD R1 and ddRAD R2 by the rare-cutting enzyme sites and the common-cutter enzyme sites, respectively..

**Cost comparison with Peterson et al. 2012 adapters and primers based on 2017 pricing from IDTechnologies.**

|  | Modified protocol | | Peterson et al. (2012) | |
| --- | --- | --- | --- | --- |
| MseI-P2.2 | $75.81 | x 1 | $72.81 | x 1 |
| MseI-P2.1 | $62.86 |  | $57.98 |  |
| EcoRI-P1.1 | $60.04 | x 1 | $59.86 | x Z |
| EcoRI-P1.2 | $82.85 |  | $81.74 |  |
| PCR1 primer | N-index $22.09 | x A | $21.62 | x 1 |
| Multiplex primer with index | S-index $23.97 | x B | $24.91 | x Y |

All costs based on 100 nM from Integrated DNA Technologies (Sept. 2017 price estimates). Cost would depend on how many indexed primers (A, B, or Y) and barcoded adapters (Z) are used. Peterson et al. (2012) and Mastretta-Yanes et al. (2015) both use 12 indexed primers (Y). Currently, there are 24 N-index and 16 S-index primers available from Nextera kit, but additional custom primers can be designed to increase multiplex capability at minimal costs. All adapters are also HPLC-purified. The primers are not HPLC-purified.

Based on the prices above and a 96-multiplex library design using 8 barcodes and 12 indexes (Peterson et al. 2012) or 8 N-indexes and 12 S-indexes, the total price of primers and adapters for the original ddRADseq comes to ($72.81 + $57.98) * 2 + ($59.86 + $81.74) * 2 * 8 + $21.62 + $24.91 * 12 = $2847.72. Our primers and adapters came to $75.81 + $62.86 + $60.04 + $82.85 + $22.09 * 8 + $23.97 * 12 = $745.92


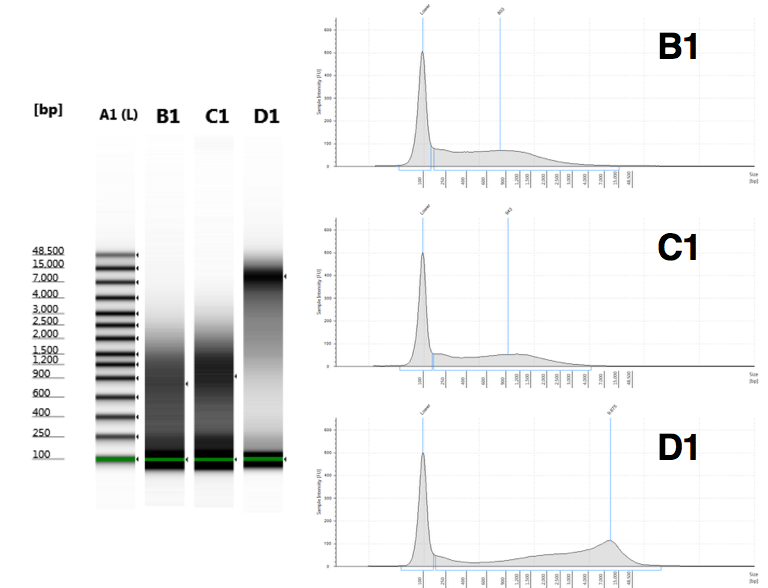


**Appendix 2** **Figure 1** Fragment analyzer results after the digestion step. Gel image (left) and intensity graphics (right). Lane A1 corresponds to the ladder; lane B1 is the double-digested sample; lane C1 is single digestion with MseI; and lane D1 is single digestion with EcoRI-HF.


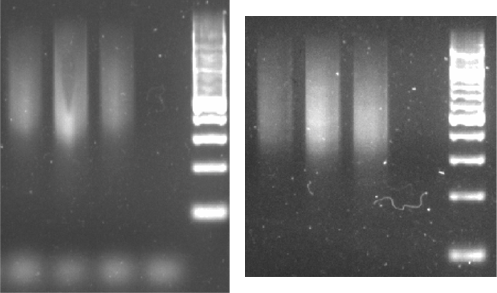


**Appendix 2** **Figure 2** Example 1% agarose gels of double-digested DNA ligated to adapters before (left) and after (right) step 3. In both cases, fourth lane corresponds to the negative control. Fifth lane is 100 bp ladder


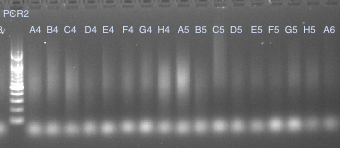


**Appendix 2** **Figure 3** An example of 1.5% agarose gel with some samples after PCR amplification (step 4). First lane is 100 bp ladder.


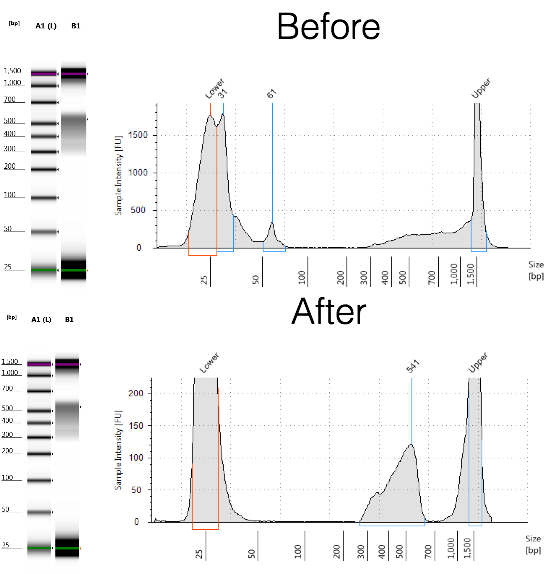


**Appendix 2** **Figure 4** TapeStation results before and after size selection (step 6).

1. Previous adaptors design match a kit no longer sold by Illumina: Multiplexing Sample Prep Oligo Only kit (see Illumina Document #1000000002694 v03 October 2017, page 35). [↑](#footnote-ref-1)
